# The complete mitogenome of the omei toothed toad, *Oreolalax omeimontis* (Anura: Megophryidae) with phylogenetic analysis

**DOI:** 10.1080/23802359.2020.1798295

**Published:** 2020-08-07

**Authors:** Mo Wang, Zhuoyun Jiang, Jishan Wang, Mingwang Zhang

**Affiliations:** a Key Laboratory for Conserving Wildlife with Small Populations in Yunnan, Faculty of Biodiversity Conservation, Southwest Forestry University, Kunming, Yunnan, PR China; b State Key Laboratory of Genetic Resources and Evolution, Kunming Institute of Zoology, Chinese Academy of Sciences, Kunming, Yunnan, PR China; c China Forest Exploration & Design Institute in Kunming, State Forestry and Grassland Administration PR China, Yunnan, PR China; d College of Animal Science and Technology, Sichuan Agricultural University, Chengdu, PR China; e Animal Genetic Resources Exploration and Innovation Key Laboratory of Sichuan Province, Sichuan Agricultural University, Chengdu, Sichuan, China

**Keywords:** complete mitogenome, *Oreolalax omeimontis*, phylogenetic analysis

## Abstract

We sequenced and annotated the complete mitogenome sequence of *Oreolalax omeimontis* (17,675 bp long). The mitogenome encoded 13 protein-coding genes (PCG), 2 ribosomal RNA (rRNA) genes, 23 transfer RNA (tRNA) genes, and a control region (GenBank accession number MN803321). The phylogenetic tree conforms the close relationship of *O. omeimontis* and *O. multipunctatus,* and a monophyletic clade of genus *Oreolalax*.

The toothed toad, genus *Oreolalax*, belonging to the family Megophryidae, comprises 19 species distributed in southwestern China and adjacent Vietnam (Frost 2020), and 18 that are distributed in China (AmphibiaChina 2020). The large toothed toad (*Oreolalax omeimontis*) is found only in montane regions of central Sichuan (Emei and Hongya Counties), China (Fei et al. 2012; Frost 2020) at the elevation from 1050 to 1800 m. Recently, Huang et al. reported a compete mitogenome of *Oreolalax major* and provided neighbor-joining tree of the *Oreolalax* (Huang et al. 2020). To date, only seven *Oreolalax* species mitogenome sequences are available from NCBI GenBank (see Figure 1). In this study, a complete mitogenome of *O. omeimontis* was sequenced, which will provide insights into mitogenome evolution and explore the phylogenetic relationship of *Oreolalax*.

**Figure 1.**
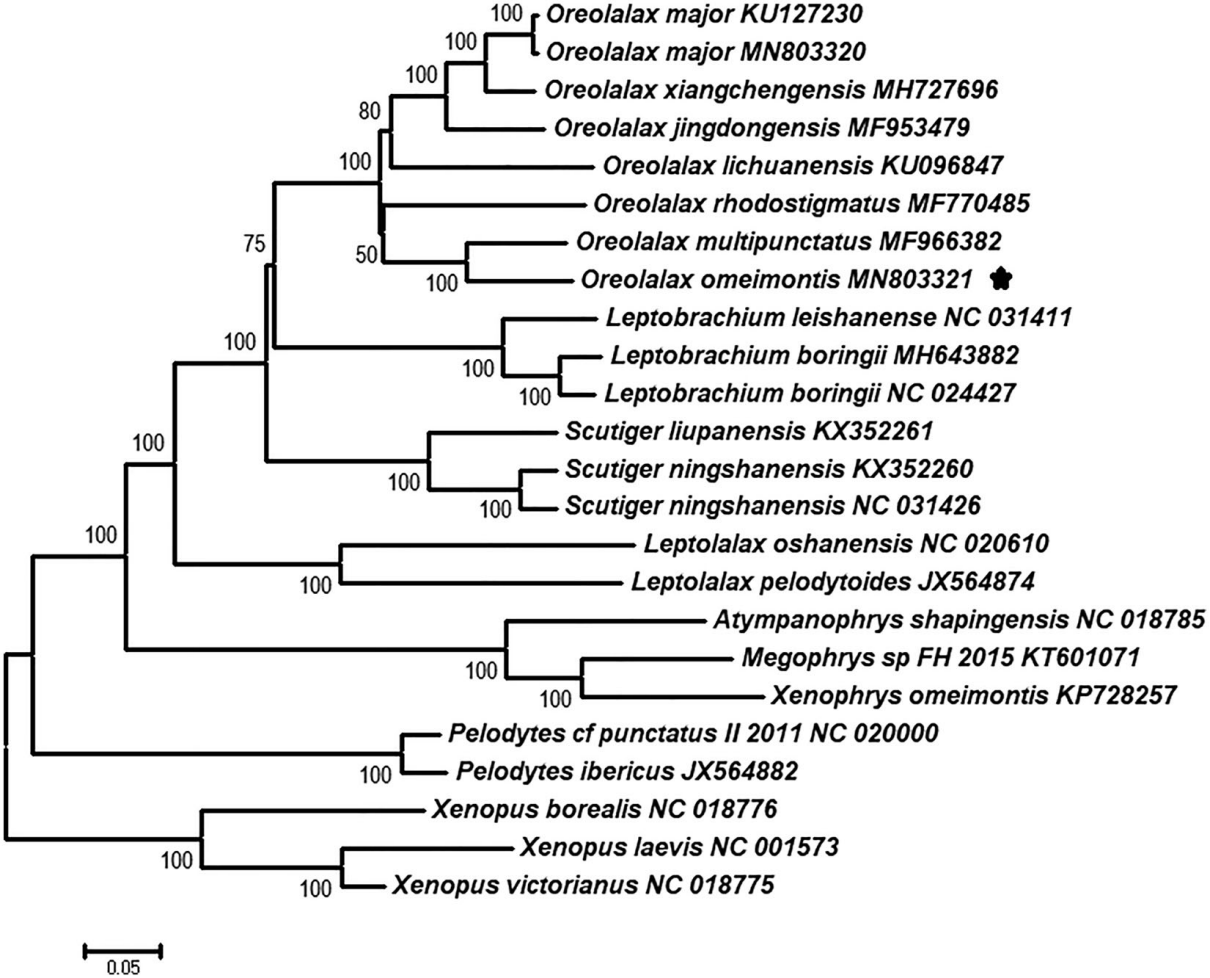
Neighbour-joining (NJ) phylogenetic tree based on all 13 combined mitochondrial protein-coding genes from 21 species. The numbers of internal branches are bootstrap values.

The specimen of *O. omeimontis* was collected from Wawushan Nature Reserve (29°40′2.89″N, 102°58′28.86″E., elev. 1400 m), Sichuan Province, China, in 2015, which was deposited in the Museum of Sichuan Agricultural University (Specimen voucher: 20120084). Total genomic DNA was extract from ethanol-preserved muscle tissue by using the Ezup pillar genomic DNA extraction kit (Sangon Biotech, Shanghai, China). The whole genome library was constructed and sequenced on an Illumina MiSeq platform (PE400) by Personal Biotechnology (Shanghai, China).

The closed-circular mitogenome (MN803321) of *O*. *omeimontis* contained 38 genes including 13 protein-coding genes (PCGs), 2 ribosomal RNA (rRNA) genes, 23 tRNA genes (including two *trnM* genes) and a control region (D-loop). The total mitogenome length of *O*. *omeimontis* is 17,675 bp, and the base composition of the mitogenome is 28.46% A, 13.99% G, 24.96% C, 32.59% T, respectively. Comparative analysis indicates that the mitogenome structure of *O. omeimontis* is similar to that of other Megophryidae (Xiang 2013; Liang 2016; Liang et al. 2016). *Nad6* and eight *tRNAs* (*tRNA-Q, A, N, C, Y, S*_2_*, E* and *P*) are located on the L-light strand (L strand), and the others are encoded on the heavy strand (H strand). Most of the PCGs (nine) were started by ATG, except two PCGS (*cox1* and *nad4l*) by GTG, and *nad3* by ATA. *Nad1* was terminated by TAG, three PCGs (*cox1, nad5* and *nad6*) terminated by AGG as termination codon, and four (*nad2*, *atp8*, *nad3* and *nad4l*) terminated by TAA. Two PCGs (*atp6* and *cox3*) stopped with TA, whereas three (*cox2*, *nad4* and *cytb*) stopped with a single base T. The length of 12S and 16S rRNA are 936 and 1589 bp respectively. The length of mitogenome ranges from 17,110 bp in *O*. *xiangchengensis* to 18,676 bp in *O. rhodostigmatus*. The small differences in size between *Oreolalax* mitogenome was largely due to an expansion of control region (Huang et al. 2020).

The final alignment consisted of 24 sequences of 21 species from nine genera (*Oreolalax, Leptobrachium, Scutiger, Leptolalax, Atympanophrys, Megophrys, Xenophrys, Pelodytes,* and *Xenopus*). Phylogenetic trees of the family Megophryidae were reconstructed by MEGA7.0 (Kumar et al. 2016) using 13 combined PCGs with three *Xenopus* species as outgroups. The close relationship of *O. omeimontis* and *O. multipunctatus,* and a monophyletic clade of genus *Oreolalax* were confirmed by 100% bootstrap support (Figure 1). More mitogenome sequences of other *Oreolalax* species are needed and that would be useful to understand molecular evolution and elucidate phylogenetic relationships among *Oreolalax*.

## Data Availability

The data used to support the findings of this study are available in MITOS WebServer at http://mitos2.bioinf.uni-leipzig.de/overview.py?hash=tZY1Qh2i or in GenBank at https://www.ncbi.nlm.nih.gov/nuccore/MN803321, reference number MN803321.
